# An allosteric binding site of the α7 nicotinic acetylcholine receptor revealed in a humanized acetylcholine-binding protein

**DOI:** 10.1074/jbc.M117.815316

**Published:** 2017-12-13

**Authors:** Florian Delbart, Marijke Brams, Fabian Gruss, Sam Noppen, Steve Peigneur, Sandro Boland, Patrick Chaltin, Jose Brandao-Neto, Frank von Delft, Wouter G. Touw, Robbie P. Joosten, Sandra Liekens, Jan Tytgat, Chris Ulens

**Affiliations:** From the ‡ Department of Cellular and Molecular Medicine, Laboratory of Structural Neurobiology, Faculty of Medicine, KU Leuven, 3000 Leuven, Belgium,; the § Department of Microbiology and Immunology, Laboratory of Virology and Chemotherapy, Rega Institute for Medical Research, KU Leuven, 3000 Leuven, Belgium,; the ¶ Laboratory of Toxicology and Pharmacology, Faculty of Pharmaceutical Sciences, KU Leuven, 3000 Leuven, Belgium,; the ‖ Center for Innovation and Stimulation of Drug Discovery Leuven, Cistim Leuven vzw, 3001 Heverlee, Belgium,; the ** Center for Innovation and Stimulation of Drug Discovery Leuven and Center for Drug Design and Discovery, KU Leuven, 3001 Heverlee, Belgium,; ‡‡ Diamond Light Source Ltd., Harwell Science and Innovation Campus, Didcot OX11 0QX, United Kingdom, and; the §§ Division of Biochemistry, Netherlands Cancer Institute, 1066CX Amsterdam, The Netherlands

**Keywords:** Cys-loop receptor, electrophysiology, neurotransmitter, nicotinic acetylcholine receptors (nAChR), X-ray crystallography, allosteric modulation, fragment-based screening, ligand-gated ion channel, structure-based drug discovery

## Abstract

Nicotinic acetylcholine receptors (nAChRs) belong to the family of pentameric ligand-gated ion channels and mediate fast excitatory transmission in the central and peripheral nervous systems. Among the different existing receptor subtypes, the homomeric α7 nAChR has attracted considerable attention because of its possible implication in several neurological and psychiatric disorders, including cognitive decline associated with Alzheimer's disease or schizophrenia. Allosteric modulators of ligand-gated ion channels are of particular interest as therapeutic agents, as they modulate receptor activity without affecting normal fluctuations of synaptic neurotransmitter release. Here, we used X-ray crystallography and surface plasmon resonance spectroscopy of α7-acetylcholine–binding protein (AChBP), a humanized chimera of a snail AChBP, which has 71% sequence similarity with the extracellular ligand-binding domain of the human α7 nAChR, to investigate the structural determinants of allosteric modulation. We extended previous observations that an allosteric site located in the vestibule of the receptor offers an attractive target for receptor modulation. We introduced seven additional humanizing mutations in the vestibule-located binding site of AChBP to improve its suitability as a model for studying allosteric binding. Using a fragment-based screening approach, we uncovered an allosteric binding site located near the β8–β9 loop, which critically contributes to coupling ligand binding to channel opening in human α7 nAChR. This work expands our understanding of the topology of allosteric binding sites in AChBP and, by extrapolation, in the human α7 nAChR as determined by electrophysiology measurements. Our insights pave the way for drug design strategies targeting nAChRs involved in ion channel–mediated disorders.

## Introduction

Cys-loop receptors, also called pentameric ligand-gated ion channels (pLGICs),^4^ are involved in fast synaptic transmission in the central and peripheral nervous systems. This channel family can be subdivided into two main subfamilies. One subfamily is formed by pLGICs that selectively permeate cations and are involved in excitatory synaptic transmission, namely nicotinic acetylcholine receptors (nAChRs) and type-3 serotonin receptors. The second subfamily is formed by pLGICs that selectively permeate anions and are involved in inhibitory synaptic transmission, namely type A γ-aminobutyric acid receptors (GABA_A_Rs) and glycine receptors (GlyRs). For recent reviews, see Refs. 1–5. Each of these pLGICs share a common assembly of five identical or non-identical subunits arranged around a central ion-conducting pore. Each subunit consists of an extracellular ligand-binding domain, a pore-forming transmembrane domain, and an intracellular domain. The neurotransmitter-binding site is located in the ligand-binding domain at the interface between each of two subunits, whereas the channel gate is at a spatially distinct, yet conformationally coupled location in the pore domain.

From a therapeutic perspective, two specific nAChR subtypes have been explored extensively, namely the homomeric α7 nAChR and the heteromeric α4β2 nAChR. The α4β2 nAChR is the target for the addictive effects of nicotine in tobacco smokers and a partial agonist, varenicline (Champix®), has reached the market for the treatment of nicotine addiction (6). The highest affinity receptors are composed of two α4 subunits and three β2 subunits and are localized in the mesolimbic dopaminergic system where stimulation of these receptors results in dopamine release, which mediates the reinforcing effects of nicotine. Varenicline is a partial agonist for α4β2 nAChRs causing partial receptor stimulation while competitively inhibiting nicotine binding (6). The α7 nAChR has attracted considerable attention to enhance cognition in neurological and psychiatric disorders associated with cognitive decline such as Alzheimer's disease and schizophrenia (7–11). Agonists for α7 nAChRs have a major disadvantage that prolonged receptor activation produces sustained receptor activation and desensitization, thereby decreasing the normal fluctuations in acetylcholine signaling at the synapse (9). In contrast, positive allosteric modulators (PAMs) of α7 nAChRs enhance receptor function in the presence of acetylcholine while preserving normal fluctuations of receptor activation (10).

Structural insight into the molecular architecture of integral nAChRs has been revealed through the pioneering work of Unwin *et al*. (12) on the *Torpedo* nAChR and recently, with the X-ray structure determination of the human α4β2 nAChR bound to nicotine (13). In contrast, a three-dimensional structure for the integral α7 nAChR is not yet available, but a wealth of structural data exist for the acetylcholine-binding proteins (AChBPs) (14, 15), which are water-soluble surrogates of the ligand-binding domain of nAChRs. At present, more than 100 crystal structures have been determined of AChBPs in complex with a wide variety of agonists, partial agonists and antagonists, as available in the Protein Data Bank. In addition, a chimera of AChBP and the extracellular ligand-binding domain human α7 nAChR, named α7-AChBP, has been engineered that has 64% sequence identity and 71% sequence similarity with the α7 nAChR ligand-binding domain (16). Previously, we have employed α7-AChBP to unveil novel allosteric binding sites using a fragment-based screening approach in which the neurotransmitter-binding site was occupied with a known partial agonist and unoccupied allosteric sites were identified through novel binders from a fragment library (17). Among the identified allosteric sites the so-called vestibule-binding site appears to be particularly attractive as it overlaps with a benzodiazepine-binding site in the prokaryote ligand-gated ion channel ELIC, which is positively modulated by the benzodiazepine flurazepam (18).

In this study, we extended these observations and engineered an improved α7-AChBP harboring additional humanizing mutations in the vestibule-binding site. Using a fragment-based screening approach we identified additional allosteric binders and unexpectedly, we unveiled a novel allosteric binding site, which is located near the β8–β9 loop. This site partially overlaps with a known modulatory site for Ca^2+^, which potentiates the human α7 nAChR (19).

## Results

### Engineering humanizing mutations in the vestibule-binding site of α7-AChBP

The aim of our study was to enhance our understanding of the structural determinants of allosteric modulation of nAChRs and to develop improved tools for structure-based design of new therapeutics targeting the human α7 nAChR. To this end, we employed α7-AChBP, which is a previously designed chimera between the human α7 nAChR and the *Lymnaea stagnalis* AChBP and has 64% sequence identity with the α7 nAChR ligand-binding domain (16). Our preferred target site for allosteric modulation of the receptor is the previously identified vestibule-binding site (17), which corresponds to a benzodiazepine-binding site of the prokaryote ligand-gated ion channel ELIC, which is positively modulated by the benzodiazepine flurazepam (18). To improve the resemblance of α7-AChBP to the human α7 nAChR we introduced additional humanizing mutations in the local environment of the vestibule-binding site based on the amino acid interactions formed with a previously identified vestibule binder, named fragment molecule 4 (PDB code 5afm) (17). In total, we expressed 12 single point mutants substituting each residue by its corresponding residue in the human α7 nAChR, namely F52I, I80D, V85K, L88I, A89L, A90L, S95E, K96R, P97F, I119G, Q121F, and F123S. Among these 12 mutants, 7 were tolerated, namely F52I, V85K, L88I, A89L, A90L, S95E, and K96R. We defined a “tolerated” mutation as a mutation that results in detectable protein secreted in the expression medium, similar to wildtype α7-AChBP. “Not-tolerated” mutations failed to produce secreted protein, because of folding, trafficking, or other issues. The remaining 5 mutants were not-tolerated. Next, the 7 tolerated mutations in the vestibule-binding site were combined into a single construct, which we termed α7-AChBP_VS_.

### X-ray crystal structure determination of ligand-bound complexes of α7-AChBP_VS_

**Figure 1.**
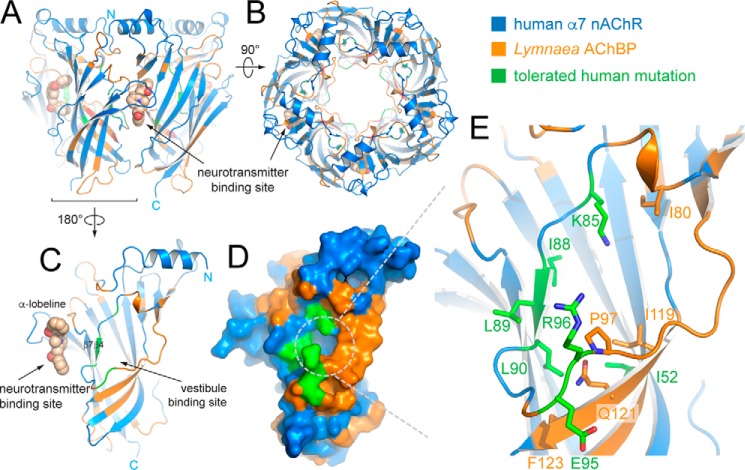
**X-ray crystal structure of α7-AChBP_VS_ in complex with α-lobeline.** *A,* schematic representation of the α7-AChBP_VS_ pentamer as seen perpendicular to the 5-fold symmetry axis. Residues belonging to the human α7 nAChR are colored in *blue* and residues from *Lymnaea* AChBP are colored *orange*. The partial agonist α-lobeline is bound to the neurotransmitter (orthosteric)-binding site of the subunit interface facing the viewer and is shown in *sphere* representation. Carbon atoms are colored in *wheat*, nitrogen in *blue*, and oxygen in *red. B,* view from (*A*) 90° rotated with the top (N-terminal side) of the pentamer facing the viewer. *C* and *D,* schematic and surface representation of a single subunit oriented toward the vestibule-binding site (180° rotated from A), which lies opposite to the neurotransmitter-binding site and is accessible via the receptor vestibule. Additional amino acid residues that were mutated into corresponding residues from the human α7 nAChR are colored in *green. E,* detailed view of the vestibule-binding site with key side chains shown in stick representation. *Orange*/*blue* residues are colored coded as in previous panels and *green* residues correspond to additional humanizing mutations.

We determined the X-ray crystal structure of α7-AChBP_VS_ in complex with the partial agonist α-lobeline (Table 2). Similar to our previous approach (17) we chose to determine structures bound to α-lobeline because crystals grown in the presence of α-lobeline gave more robust diffraction than apo crystals, thus facilitating subsequent studies with allosteric binders. Fig. 1, *A* and *B*, shows the overall architecture of lobeline-bound α7-AChBP_VS_, which is composed of 5 subunits radially arranged around a central pore. Human α7 nAChR residues are colored in *blue* and *Lymnaea* AChBP residues are colored in *orange*. The neurotransmitter-binding site is localized at the interface between each of two subunits and is occupied by α-lobeline in this structure (shown in *sphere* representation in Fig. 1). Fig. 1, *C* and *D*, shows a single α7-AChBP_VS_ subunit and the location of the vestibule-binding site relative to the neurotransmitter-binding site. The vestibule-binding site is an intrasubunit site accessible from the receptor vestibule and located opposite to the neurotransmitter-binding site. Both sites are separated by a wall including the β4- and β7-strands. As can be seen from the surface representation in Fig. 1*D* the amino acid residues composing the vestibule-binding site now have an improved identity with human α7 nAChR residues (colored in *green*). In α7-AChBP_VS_ the local environment of the vestibule-binding site was partially humanized through 7 mutations, F52I, V85K, L88I, A89L, A90L, S95E, and K96R (shown in *green sticks* in Fig. 1*E*).

**Figure 2.**
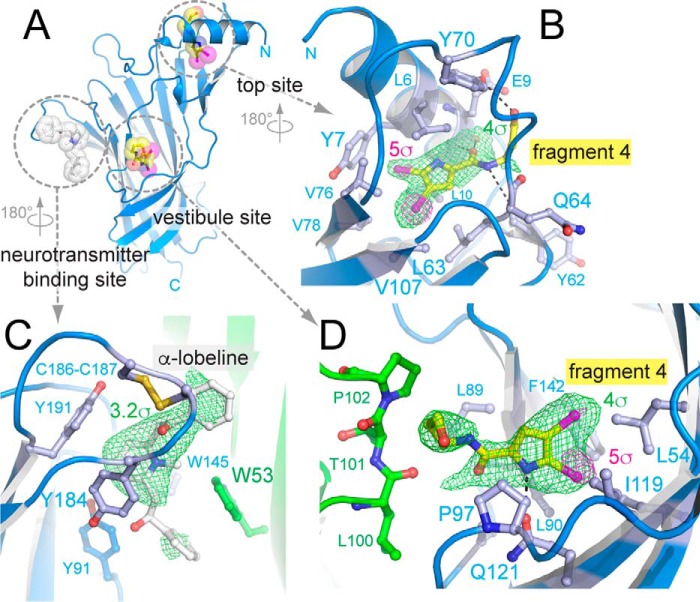
**X-ray crystal structure of α7-AChBP_VS_ in complex with α-lobeline and allosteric binder fragment 4.** *A,* schematic representation of a single α7-AChBP_VS_ subunit as seen toward the vestibule site. α-Lobeline is bound to the neurotransmitter-binding site and is shown in *white transparent* sphere and stick representation. Fragment 4 is bound to two different allosteric sites, the vestibule site and the top site and is shown in *yellow* (carbon), *blue* (nitrogen), *red* (oxygen), and *magenta* (bromine). *B,* detailed view of the amino acid interactions formed by fragment 4 at the top site. The *green mesh* is the 5-fold averaged simple difference density at a contour level of 4σ, the *magenta mesh* is the anomalous difference density at 5σ. *C,* detailed view of amino acid interactions formed by α-lobeline at the neurotransmitter binding site. The principal subunit is shown in *blue*, the complementary subunit in *green*. The highly conserved aromatic side chains are shown as ball and sticks. The *green mesh* is the 5-fold averaged simple difference density at 3.2σ. *D,* detailed view of amino acid interactions formed by fragment 4 at the vestibule site. Most interactions are intrasubunit interactions (*blue*), whereas 3 residues come from a neighboring subunit (*green*). The *green mesh* is the 5-fold averaged simple difference density at a contour level of 4σ, the *magenta mesh* is the anomalous difference density at 5σ.

To confirm the structural integrity of the vestibule-binding site in α7-AChBP_VS_ we also determined the X-ray crystal structure of α7-AChBP_VS_ in complex with α-lobeline and the allosteric binder fragment 4 (Tables 1 and 2). Similar to our previous approach (17) we took advantage of the 2 bromine atoms in fragment 4 to pinpoint their location in an anomalous density map. Fig. 2, *A–D*, shows a schematic representation of a single α7-AChBP_VS_ subunit and ligands shown in ball and stick representation. Unexpectedly, we observe that fragment 4 not only occupies the vestibule-binding site, but also another allosteric site at the N-terminal top side of the receptor, which we termed the top binding site (Fig. 2, *A* and *B*) as it was also bound by a different fragment molecule 1 in our previous study (17). Inspection of the anomalous density map reveals peaks (shown as magenta mesh in Fig. 2, *B* and *D*) almost twice as intense at the top binding site compared with the vestibule-binding site suggesting that fragment 4 even binds the top site with higher occupancy than the vestibule site. The fact that we observe fragment 4 not only in the vestibule site, but also in the top site could be explained by possible allosteric effects of the humanizing mutations on the top site, or alternatively, by the slightly differing crystallization conditions for the α7-AChBP_VS_ complex compared with α7-AChBP in our previous study (17). In the top site, the carboxamide moiety of fragment 4 forms hydrogen bonds (indicated as *black dashed lines* in Fig. 2*B*) with the backbone atoms of Glu-9, Leu-10, and Tyr-62, whereas the side chain of Leu-10 flips outward to expose the binding site. The hydroxypropyl moiety of fragment 4 forms a hydrogen bond with Tyr-70, which is located in a loop connecting the β2- and β3-strands. Additionally, fragment 4 forms van der Waals interactions with Leu-6, Tyr-7, Leu-63, Gln-64, Val-76, Val-78, and Val-107.

**Table 1:** **Crystallographic and refinement statistics** Resolution cut-off criteria were 〈*I*/σ〉 ≥ 1.0 and CC1/2 ≥ 30% (42). Values between parentheses are for the highest resolution shell.

Crystallographic statistics	α7-AChBPvs +α-lobeline	α7-AChBPvs +α-lobeline and fragment 4	α7-AChBPvs +fragment CU2017
PDB accession code	5ouh	5oug	5oui
Beamline	I04-1 (DLS)	PROXIMA-I (SOLEIL)	I04–1 (DLS)
Date of collection	15-Mar-2017	10-Feb-2017	16-Mar-2017
Wavelength (Å)	0.92818	0.919830	0.92818
Space group	*P*2_1_2_1_2_1_	*P*2_1_	*P*2_1_2_1_2_1_
*a, b, c* (Å)	87.79, 113.32, 146.96	68.673, 119.805, 84.543	87.75, 113.14, 145.89
α, β, γ (°)	90.00, 90.00, 90.00	90.000, 107.288, 90.000	90.00, 90.00, 90.00
Resolution limits (Å)	87.79 - 2.50 (2.58 - 2.50)	66.95 - 2.57 (2.67 - 2.57)	72.94 - 3.10 (3.29 - 3.10)
*R*_merge_	14.1 (289)	12.8 (213)	50.4 (445)
*R*_meas_	15.2 (313)	15.3 (253)	54.4 (480)
*R*_pim_	5.7 (119)	8.3 (135)	20.3 (179)
〈*I*/σ〉	12.7 (1.0)	11.4 (1.3)	6.4 (1.0)
CC1/2 (%)	99.9 (54.3)	99.6 (30.5)	98.3 (43.0)
Multiplicity	13.5 (13.3)	6.7 (6.5)	13.4 (13.6)
Completeness (%)	100.0 (100.0)	99.8 (98.6)	100.0 (100.0)
Total number of reflections	695,420 (59,142)	277,801 (31,273)	362,469 (58,442)
Number of unique reflections	51,511 (4,447)	41,656 (4,603)	27,070 (4,285)
Anomalous completeness		98.6 (97.4)	99.9 (100.0)
Anomalous multiplicity		3.4 (3.4)	7.1 (7.1)
Refinement and model statistics			
*R*_work_ (%)	20.7 (36.9)	23.1 (36.6)	24.1 (38.8)
*R*_free_ (%)	25.0 (36.3)	26.0 (33.2)	28.0 (50.2)
Bond length root mean square deviation (Å)	0.009	0.008	0.010
Bond angle root mean square deviation (°)	1.400	1.349	1.486
Ramachandran plot favored/outliers (%)	98.13/0	98.72/0	98.33/0
Rotamer favored/outliers (%)	96.36/0	95.52/0.2	93.66/1.04
MolProbity score (percentile)	2.39 (100th percentile)	1.00 (100th percentile)	1.61 (100th percentile)

**Table 2:**
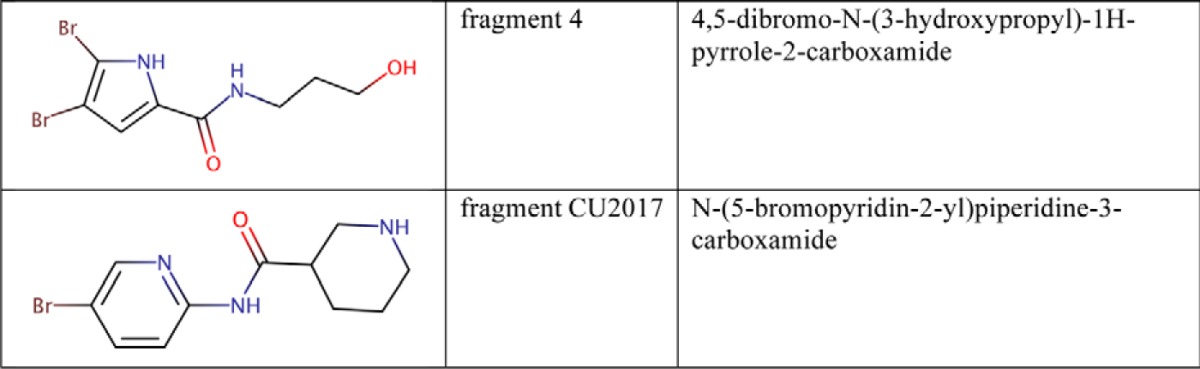
**Chemical structures of allosteric fragment molecules**

In the vestibule-binding site (Fig. 2*D*) fragment 4 forms a hydrogen bond (indicated as *black dashed line*) via the pyrrole nitrogen and Gln-121. Van der Waals interactions are formed with Leu-54, Leu-89, Leu-90, Pro-97, Leu-100, Thr-101, Pro-102, Ile-119, and Phe-142.

In the neurotransmitter-binding site (Fig. 2*C*) the difference density map reveals features for the central piperidine moiety of α-lobeline, but less clearly for the extreme phenyl moieties. This can be explained by the fact that α-lobeline is flexible and adopts different partially overlapping ligand binding poses around the piperidine moiety as observed in previous structures of *Aplysia* and *Capitella* AChBPs and α7-AChBP (20, 21).

### Characterization of the binding properties of α7-AChBP_VS_ via surface plasmon resonance spectroscopy

To further validate α7-AChBP_VS_ as an improved model for structural and functional studies we verified the pharmacological properties of α7-AChBP_VS_ via surface plasmon resonance (SPR) spectroscopy. We determined the binding characteristics of a range of representative agonists (acetylcholine, nicotine), partial agonists (varenicline, α-lobeline), antagonists (*d*-tubocurarine, strychnine), and allosteric modulators (fragment 4 and additional binders described below) and compared them for α7-AChBP_VS_ and α7-AChBP (17).

Overall, binding affinities of the tested ligands were comparable for α7-AChBP_VS_ and α7-AChBP with the exception of acetylcholine, varenicline, and nicotine, which displayed 10-fold higher *K_D_* values for α7-AChBP_VS_ compared with α7-AChBP (Table 3). Although the neurotransmitter-binding site was preserved from mutations, these orthosteric binders showed a lower affinity, which was not observed with the other compounds that bind the same pocket. The mutations in the vestibule-binding site seemed not to alter the binding affinity for the previously described allosteric binder, fragment 4. Both proteins showed a weak interaction with fragment 4 with a *K_D_* of 1420 μm for α7-AChBP and 1703 μm for α7-AChBP_VS_. However, the latter represents an “apparent” *K_D_* value because it is higher than half the highest analyte concentration used (*i.e.* 3 mm). Together, these results validate α7-AChBP_VS_ as an improved model for structural and functional studies of ligand recognition at the vestibule-binding site. Because the additional humanizing mutations appear to affect the orthosteric binding site it is important to interpret results related to this site with caution.

**Table 3:** **Equilibrium dissociation constants (*K_D_*) as determined for different ligands via surface plasmon resonance spectroscopy** Data are reported as average ± S.D. of at least 4 experiments (*, **, and *** indicate significant difference at *p* values of 0.05, 0.01, and 0.001, respectively).

	*K_D_* α7-AChBP	*K_D_* α7-AChBP_VS_	
	μ*m*		
Acetylcholine	23.92 ± 4.83	202.38 ± 81.32	***
Nicotine	1.60 ± 0.19	11.40 ± 0.80	***
Varenicline	1.24 ± 0.17	9.90 ± 1.28	***
α-Lobeline	0.75 ± 0.13	2.14 ± 1.06	*
*d*-Tubocurarine	1.29 ± 0.19	1.41 ± 1.02	NS*^a^*
Strychnine	2.14 ± 0.51	2.64 ± 0.54	NS
Fragment 4	1420.00 ± 98.49	1703.44 ± 627.74	NS
CU2017	40.04 ± 19.47	93.57 ± 35.08	**

*^a^* NS, not significant.

### Deployment of α7-AChBP_VS_ for high-throughput data collection of fragment libraries

Our next goal was to obtain additional fragment molecules binding the vestibule site as a template to design a high affinity lead compound targeting this site. A conventional approach is to pre-screen fragment hits from a fragment library using a biophysical method amenable to high-throughput screening, such as SPR, and to select those hits for structure determination by X-ray crystallography. However, example cases have shown that hit selection highly depends on the biophysical screening method used and consequently, a large proportion of fragment molecules suitable for structure determination can be missed (23). With the recent development of automated data collection pipelines at synchrotron beamlines data sets of an entire fragment library can now be collected in 1 week, thus eliminating the need to employ a pre-screening selection.

To this end, we employed AChBP_VS_ at such a beamline, the XChem facility of the Diamond Light Source (Oxfordshire, UK). As part of a test run we collected structures for a partial library composed of 115 fragments. To further advance method development at automated synchrotron beamlines we investigated the usefulness of PDB-REDO (see “Experimental procedures”), which is an automated procedure to optimize macromolecular structure models (24, 25). We found that PDB-REDO improves model *R*_free_ factors by 1.6% on average (minimum 0% and maximum 4.6%), thus contributing to model improvement and better electron density maps for interpretation of fragment hits. From this set we could identify 2 hits, one is localized in the vestibule-binding site, thus confirming our earlier observations of the importance of this site (17). A second hit is localized in a novel allosteric binding site in α7-AChBP_VS_ as discussed below.

### Identification of an allosteric binding site near the β8–β9 loop of α7-AChBP_VS_

**Figure 3.**
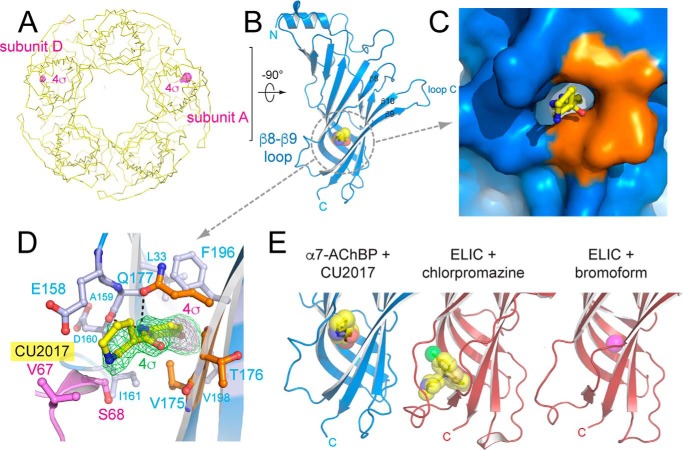
**X-ray crystal structure of α7-AChBP_VS_ in complex with allosteric binder fragment CU2017.** *A, yellow ribbon* representation of the α7-AChBP_VS_ pentamer as seen along the 5-fold symmetry axis with the bottom (C-terminal side) of the pentamer pointing toward the viewer. The *magenta mesh* is the anomalous difference density at a contour level of 4σ. *B,* schematic representation of the A subunit with fragment CU2017 bound to the β8–β9 loop site. Fragment CU2017 is shown in transparent ball & stick representation in *yellow* (carbon), blue (nitrogen), *red* (oxygen), and *magenta* (bromine). *C* and *D,* detailed surface and ball and stick representation of the β8–β9 loop site. Residues belonging to the human α7 nAChR are colored in *blue* and residues from *Lymnaea* AChBP are colored in *orange*. Residues Val-67 and Ser-68 (*pink*) are from a neighboring pentamer in the crystal packing. In *D,* the *green mesh* is the simple difference density at a contour level of 4σ, the *magenta mesh* is the anomalous difference density at 4σ. *E,* comparison of the β8–β9 loop site occupied by fragment CU2017 and previously published structures of the *Erwinia* ligand-gated ion channel ELIC in complex with chlorpromazine (28) (PDB code 5lg3) or bromoform (27) (PDB code 3zkr).

From our fragment screen we identified a molecule termed CU2017 ((*N*-(5-bromopyridin-2-yl)piperidine-3-carboxamide) (Tables 1 and 2), which occupies a novel binding site located near the β8–β9 loop (Fig. 3, *A–D*). In integral pLGICs this loop contributes to the interface with the transmembrane domain and plays an important role in coupling ligand binding to channel opening. CU2017 contains a single bromine atom and the observed anomalous signal unambiguously pinpoints the location of this fragment molecule. Fig. 3*A* shows two peaks in the anomalous density map, contoured at a level of 4σ, and visible in subunits A and D. The difference density is only sufficiently clear to allow ligand building in subunit A, suggesting that the occupancy in subunit D is lower. The β8–β9 site occupied by CU2017 is mainly composed of human α7 amino acid residues (indicated in *blue* in Fig. 3, *C* and *D*) and only 3 residues from *Lymnaea* AChBP remain, namely Val-175, Thr-176, and Gln-177 (indicated in *orange* in Fig. 3, *C* and *D*). The bromo-pyridine moiety of CU2017 points deep into the β8–β9 site, whereas the piperidine moiety points outward. Hydrogen bonds (shown as *dashed lines* in Fig. 3*D*) are formed between the carboxamide moiety and Ala-159 and Gln-177. Van der Waals interactions are formed with Leu-33, Glu-158, Asp-160, Ile-161, Val-175, Thr-176, Phe-196, and Val-198. Additionally, interactions are formed with interacting residues from a neighboring pentamer in the crystal packing, Val-67 and Ser-68, and it is likely that these residues contribute to added ligand stability in the A subunit. The anomalous peak in subunit D indicates that CU2017 also binds in the β8–β9 site in the absence of crystal contacts albeit at lower occupancy. Importantly, the β8–β9 site in α7-AChBP_VS_ is very near to the allosteric binding site previously identified in the ELIC + bromoform (27) and ELIC + chlorpromazine structures (28) (Fig. 3*E*). This result demonstrates that the β8–β9 site is common to different pLGICs and can be commonly targeted by chemically diverse molecules in these different pLGICs.

### Electrophysiological characterization of novel fragment hits on human α7 nAChRs

**Figure 4.**
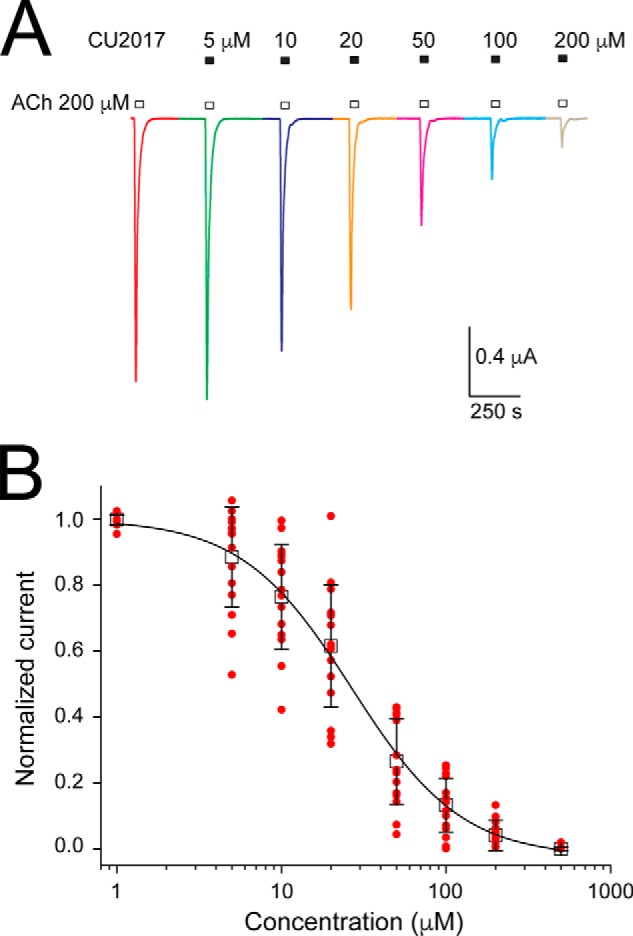
**Electrophysiological characterization of allosteric fragment CU2017 on human α7 nAChRs expressed in *Xenopus* oocytes.** *A,* agonist-evoked responses of human α7 nAChRs using 200 μm acetylcholine, first applied alone and then co-applied with increasing concentrations of CU2017 (5–200 μm). *B,* plot of the peak current responses as a function of fragment concentration and a curve fit of the data with the Hill equation. Data are shown as a scatter plot (*red circles*) and the mean ± S.D. of 16 independent experiments. The Hill plot has an IC_50_ value of 26.7 ± 1.6 μm and a coefficient of 1.3 ± 0.1 (*n* = 16).

We previously demonstrated that fragment 4, a vestibule site binder, acts as a negative allosteric modulator on the human α7 nAChR (17). In an earlier study, we demonstrated that the benzodiazepine flurazepam acts as a PAM through the vestibule-binding site of the prokaryote ligand-gated ion channel ELIC (18). To investigate the functional properties of fragment CU2017 we tested its effects on the human α7 nAChR using voltage-clamp recordings in *Xenopus* oocytes. The fragment was tested in a concentration range between 1 and 500 μm and each concentration was applied either alone or in combination with 200 μm acetylcholine (Fig. 4). When CU2017 was applied in the absence of acetylcholine, no significant effect was observed. Upon co-application with acetylcholine we observed that CU2017 causes a concentration-dependent inhibition of acetylcholine-induced responses of the α7 nAChR (Fig. 4) with an IC_50_ value of 26.7 ± 1.6 μm. Together, these data demonstrate that the fragment hit CU2017 is functionally active and inhibits channel opening likely through its corresponding site as identified in the α7-AChBP_VS_ crystal structure. Fragment CU2017 binds at the β8–β9 site and functionally behaves as a negative modulator, similar to chlorpromazine (28) and bromoform (27), which bind at an overlapping site in ELIC. Importantly, the β8–β9 site is near to a known modulatory site for Ca^2+^ potentiation in the human α7 nAChR (19).

Together, these results demonstrate that fragment-based screening identifies functionally active allosteric binders of the human α7 nAChR. As mentioned in our previous work (17), we hypothesize that chemical optimization of our fragments, increasing their molecular weight, potency, and number of interactions, could lead to a modification in functional activity and cause positive allosteric modulation of the α7 nAChR, which is therapeutically desirable for cognition enhancement (7–11).

## Discussion

PAMs of α7 nAChRs are currently investigated as promising therapeutic agents to enhance cognitive function in Alzheimer's disease and schizophrenia. Several candidate molecules have been developed (29–33) and they are broadly categorized into two classes, namely type I PAMs, which increase the peak current response and type II PAMs, which increase peak current response in combination with a profound slowing of the desensitization kinetics (34). Some PAMs, such as galanthamine (35), physostigmine (36), and NS-1738 (37) exert their effect via a binding site located in the extracellular ligand-binding domain, whereas other PAMs, such as PNU-120596 (37, 38), LY-2087101 (38), and ivermectin (39) bind at a transmembrane site. Insight into the molecular determinants of allosteric binding sites mostly derives from studies employing homology modeling, docking simulations, subunit chimeras, site-directed mutagenesis, and photolabeling (reviewed in Ref. 40). Among the best studied allosteric binding sites is the binding site for the type I PAM NS-1738 and the type II PAM PNU-120596, which share a common or overlapping binding site in an intrasubunit transmembrane cavity located between TM1 and TM4 transmembrane domains (38, 40, 41).

Structural insight into the molecular recognition of allosteric modulators of nAChRs is sorely lacking, mostly due to the limited availability of high-resolution structural data for integral nAChRs. Recently, the X-ray crystal structure was determined for the human α4β2 nAChR bound to nicotine (13) and more nAChR structures will likely become available in the near future. Additionally, a wealth of structural data already exists for a wide range of nAChR agonists, partial agonists, and antagonists bound to the orthosteric binding site in AChBP (reviewed in Ref. 3), which is a structural and functional surrogate of the nAChR extracellular ligand-binding domain, but lacks the transmembrane domain (14). Whereas the available structural data focus on the molecular determinants of ligand recognition at the orthosteric binding site, we have recently begun to explore allosteric binding sites in the α7-AChBP, a chimera of AChBP and the human α7 nAChR extracellular ligand-binding domain (16). Using a screening strategy in which the orthosteric binding site was bound by a known partial agonist (α-lobeline) we unveiled allosteric binding sites via fragment molecules from a fragment library (17). X-ray crystal structures from these fragment hits then revealed the molecular topology of allosteric binding sites in α7-AChBP (17).

**Figure 5.**
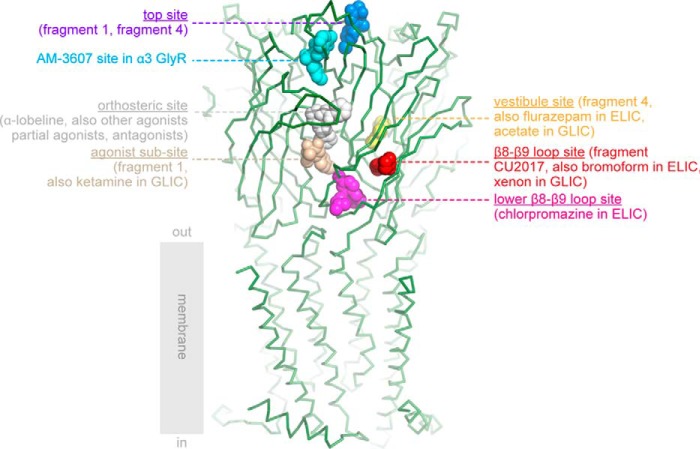
**Overview of ligand-binding sites in α7-AChBP and ligand-binding domains of other Cys-loop receptors.** The crystal structure of α7-AChBP_VS_ is shown in *green ribbon* representation and was superposed onto the extracellular domain of the human α4β2 nAChR structure (13) (PDB code 5kxi). Only the transmembrane domain of the α4β2 receptor is shown for illustrative purposes. The different binding sites are indicated according to their respective bound ligands in different Cys-loop receptor structures and corresponding PDB accession codes are mentioned between parentheses: top site in *blue*, α7-AChBP + fragment 1 (17) (code 5afj) and α7-AChBP_VS_ + fragment 4 (this study) (code 5oug); AM-3607 site in cyan, α3 GlyR + AM-3607 (43) (code 5tin); orthosteric site in *white*, example α7-AChBP_VS_ + α-lobeline (code 5ouh); agonist subsite in *wheat*, α7-AChBP + fragment 1 (17) (code 5afj) and GLIC + ketamine (49) (code 4f8h); β8–β9 loop site in *red*, α7-AChBP_VS_ + fragment CU2017 (this study) (code 5oui), ELIC + bromoform (27) (code 3zkr) and locally closed GLIC + xenon (47) (code 4zzb); lower part of the β8–β9 loop site in *magenta*, ELIC + chlorpromazine (28) (code 5lg3); vestibule site in *yellow*, AChBP_VS_ + fragment 4 structure (this study) (code 5oug), ELIC + flurazepam (18) (code 2yoe), and GLIC + bromoacetate (44) (code 4qh1).

In the present study, we expanded on these observations and engineered α7-AChBP_VS_, an α7-AChBP with additional humanizing mutations in one of the allosteric binding sites, known as the vestibule-binding site (17). Employing the latest advancements in structural methods, which allow automated data collection of thousands of fragment-soaked crystals at synchrotron radiation sources, we identify additional allosteric binders in α7-AChBP_VS_. One of these binders is fragment molecule CU2017, which binds at a novel site near the β8–β9 loop and forms part of the interface region with the transmembrane domain. In the context of the present study we discuss the relationship between allosteric binding sites in α7-AChBP and other Cys-loop receptor structures (Fig. 5). For a more extended discussion of ligand-binding sites in closed, open, and desensitized Cys-loop receptor structures we refer to a previous study (28).

The vestibule-binding site in α7-AChBP_VS_ (in *yellow*, Fig. 5) has now become one of the best characterized allosteric sites in the extracellular domain of Cys-loop receptors. The vestibule-binding site was first discovered as a benzodiazepine-binding site in the *Erwinia* ligand-gated ion channel ELIC, which is positively modulated by the benzodiazepine flurazepam (18). Later, the vestibule site was also revealed in α7-AChBP, where it was bound by fragment molecule 4 (17) and in the *Gloeobacter* ligand-gated ion channel GLIC, where it was bound by acetate (44). Very recently, the vestibule site was also identified as a site of positive allosteric modulation in a newly discovered γ-proteobacterial channel (45). In human α7 nAChRs, fragment 4 behaves as a negative allosteric modulator, whereas positive allosteric modulators are desirable to obtain cognitive enhancement in neurological and psychiatric disease (7–11). However, as exemplified in a recent study of TQS-derived allosteric modulators on human α7 nAChRs, it is possible that small changes in the chemical structures, such as methylation of an aromatic ring, can alter pharmacological profiles between PAMs and negative allosteric modulators (46). Therefore, we hypothesize that the available fragment hits for the vestibule site in the human α7 nAChR can serve the design of high affinity lead compounds targeting this site.

Fragment molecule CU2017 binds to a binding site near the β8–β9 loop in α7-AChBP_VS_ (in *red*, Fig. 5). This site was not revealed in α7-AChBP structures before, but it is near a Ca^2+^-binding site known to potentiate α7 nAChRs as identified in a mutagenesis study (19). The β8–β9 loop site is also near a binding site in ELIC bound to the antipsychotic compound chlorpromazine (28) (in *magenta*, Fig. 5) or the general anesthetic derivative bromoform (27), as well as in a locally closed form of GLIC bound to xenon (47). This site was extensively characterized in ELIC using site-directed mutagenesis and a series of phenothiazine analogues (28), which act as negative allosteric modulators of ELIC.

In this study, we observed that fragment molecule 4 also bound a binding site near the N-terminal α-helix, named the top site (in *blue*, Fig. 5), and which was also bound by fragment molecule 1 in our previous study (17). Relatively near to the top site is a novel intersubunit-binding site in the α3 glycine receptor, which is bound by the positive allosteric modulator AM-3607 (43) (in *cyan*, Fig. 5).

Just below the agonist-binding site is the so-called agonist subsite (in *wheat*, Fig. 5), which is occupied by fragment molecule 1 in α7-AChBP (17) and by ketamine in GLIC (49). Finally, there are a number of monovalent and divalent cation-binding sites in ELIC (50) and GLIC (51, 52), but which are not common with α7-AChBP and therefore not shown in Fig. 5.

Taken together, our study contributes to understanding the topology of allosteric binding sites in α7-AChBP and by extrapolation how those sites mediate allosteric modulation of the integral human α7 nAChR. An increasing number of allosteric binding sites can now be probed by chemically diverse molecules and comparison of available structures across the Cys-loop receptor family now indicates that different receptor subtypes often share common allosteric sites. The detailed structural architecture of allosteric sites, such as the vestibule site and the β8–β9 loop site, however, differs in different receptor subtypes raising the possibility to modulate receptor function with high subtype specificity. This offers new opportunities in the development of novel therapeutics targeting nAChRs in channel-related disorders.

## Experimental procedures

### Protein expression and crystallization

α7-AChBP was expressed and purified as previously described (17). In brief, α7-AChBP was expressed as a C terminally His-tagged fusion in *Sf*9 insect cells using the Bac-to-Bac baculovirus expression system from Invitrogen. Mutants were introduced using the QuikChange mutagenesis kit (Agilent) and cDNA sequences were confirmed by sequencing (LGC Genomics). At least 2 independent bacmid clones were transfected and amplified for each mutant. Each mutant was affinity-purified in batches using Ni-Sepharose High Performance beads (GE Healthcare). A final polishing purification was done on a Superdex 200 10/300 GL gel filtration column (GE Healthcare) in buffer containing 20 mm Tris, pH 8.0, 300 mm NaCl. Fractions corresponding to pentameric AChBP were loaded on SDS-PAGE for a final quality control, pooled, and concentrated to 6 mg/ml.

Concentrated protein was mixed with α-lobeline hydrochloride (Sigma) at a final concentration of 1 mm. For the complex with fragment 4, we additionally added 4,5-dibromo-*N*-(3-hydroxypropyl)-1*H*-pyrrole-2-carboxamide (Matrix Scientific) dissolved in DMSO at a final concentration of 10 mm. Crystallization plates were set up with a nanoliter Mosquito crystallization robot (TTP Labtech) and 2- or 3-well MRC plates at room temperature. Crystals grew in a variety of conditions and the best-diffracting crystals were obtained in a solution containing 0.1 m HEPES, pH 7.5, 8% ethylene glycol, and 10% PEG8000. Crystals grew to their maximal size in a few days and were harvested after cryo-protection by increasing the ethylene glycol concentration in the mother liquor to 30% in 5% increments. Crystals were then immersed in liquid nitrogen. For fragment CU2017 (Enamine), crystals were soaked at the XChem facility of the Diamond Light Source (Oxfordshire, UK) using acoustic droplet ejection with the Echo 550 Liquid Handler (Labcyte) and a fragment solution of 100 mm in DMSO.

### Structure determination

The X-ray diffraction data set for α7-AChBP_VS_ in complex with α-lobeline and fragment 4 was collected at the PROXIMA-1 beamline of the SOLEIL synchrotron (Gif-Sur-Yvette, France) at bromine inflection point wavelength of 0.91983 Å. All other data sets were collected at the I04-1 beamline of the Diamond Light Source (Oxfordshire, UK) at a fixed wavelength of 0.92 Å. Data integration and reduction were done either with XDS (53) and SCALA (54), or in the xia2 pipeline (55). The structure was solved by molecular replacement using Molrep (54) and the coordinates of α7-AChBP with PDB accession code 5afm from which ligands were removed. The raw molecular replacement solution was fed into the PDB-REDO server (24, 25) and an automated solution was obtained with refinement statistics (*r* = 22.9% and *R*_free_ = 25.2%) that outperformed a parallel effort using several cycles of manual rebuilding and refining in Coot (56). Consequently, all subsequent data sets collected at the I04-1 beamline of the Diamond Light Source were processed automatically by extending the existing DIMPLE pipeline (54) with PDB-REDO as described below. Structure validation was done using MolProbity (57) and all figures were prepared using PyMOL (Schrödinger). Anomalous maps were calculated in Phenix (58) and cut to a resolution of 5 Å for the CU2017-bound structure to improve the signal-to-noise ratio. NCS-averaged maps were calculated in Coot (56).

### Model optimization by PDB-REDO

The structure models were processed by PDB-REDO 7.00 (24, 25). The PDB-REDO pipeline consists of over 50 components that perform model optimization tasks such as (parameterization of) reciprocal- and real-space refinement, side chain rebuilding, peptide-plane flips, water removal, refinement of zinc-binding sites, carbohydrate linkage correction, twinning detection, the selection of the high-resolution data threshold, model validation, and precalculating molecular graphics scenes (59–62). All of the tasks are embedded within a fully automated decision-making framework. Recently, homology-based hydrogen-bond restraints were introduced in PDB-REDO. Model geometry and the fit to the crystallographic data improve when these restraints are imposed during model refinement, in particular at low resolution but also at intermediate and higher resolution (48). Homology restraints are particularly useful for (fragment-based) ligand screening as the proteins are identical but the data sets can vary much in resolution.

To improve deployability in high-throughput computing environments self-contained Docker (www.docker.com)^5^ and Singularity (26) images were created. The PDB-REDO Singularity image without the components that generate visualization scenes was prepared for fragment screening of α7-AChBP_VS_ at the Diamond Light Source and deployed on the High Performance Computing system brEniac of the VSC (Vlaams Supercomputing Centrum) at KU Leuven. This is a cluster consisting of 580 nodes, each equipped with a dual-socket Intel Broadwell E5–2680v4 (2.40 GHz) and 128 GB RAM. 39 useful diffraction data sets with a resolution cut-off between 2.5 and 3.5 Å were processed in less than 10 h. All models were refined in a parallel fashion with one CPU-thread per crystallographic dataset. This approach reduces the computing time of *n* datasets to the equivalent of a single dataset (∼10 h) as long as sufficient computing resources are available. Docker and Singularity PDB-REDO images are available from WGT and RPJ upon request.

Coordinates and structure factors were deposited in the Protein Data Bank under accession codes 5oug for α7-AChBP_VS_ in complex with α-lobeline and fragment 4, 5ouh for α7-AChBP_VS_ in complex with α-lobeline, and 5oui for α7-AChBP_VS_ in complex with fragment CU2017.

### Surface plasmon resonance spectroscopy

Interaction studies were performed on a Biacore T200 instrument (GE Healthcare). Proteins were covalently immobilized on a CM5 sensor chip in 10 mm HEPES using standard amine coupling chemistry. Chip densities varied between 1000 and 7000 resonance units. Interactions were performed at 25 °C in PBS-p+ (GE Healthcare) supplemented with 1% DMSO. Sample dilution series were injected for 30 s at a flow rate of 30 μl/min followed by a dissociation phase of 4 min. No regeneration of the surface was needed. A reference flow was used as a control for nonspecific binding and refractive index changes. Several buffer blanks were used for double referencing and a DMSO concentration series was included to remove the DMSO effect on the measured responses. 50 μm *d*-tubocurarine was used as positive control. Equilibrium dissociation constants (*K_D_*) were calculated in the Biacore T200 Evaluation Software 2.0.

Because of the characteristic fast binding kinetics of small molecules a steady state analysis was applied to calculate the binding affinities. The binding levels at equilibrium (*R*_eq_) were plotted against the corresponding analyte concentrations (*C*) and the equilibrium dissociation contant (*K_D_*) was derived from *R*_eq_ = *CR*_max_/(*K_D_* + *C*). *R*_max_ represents the analyte binding capacity of the surface.

### Electrophysiological recordings

For expression in *X. laevis* oocytes, the plasmid encoding the human α7 nAChR subunit (α7 subcloned in vector pMXT) was linearized with BamHI and capped cRNAs were synthesized from the linearized plasmids by using the SP6 mMESSAGE-mMACHINE transcription kit (Ambion). Stage V and VI oocytes were harvested from the ovarian lobes of anesthetized frogs as described (22). Freshly isolated oocytes were subsequently injected with 45 nl of cRNA at a concentration of ∼100 ng/μl by using a microinjector (Drummond). The oocyte incubation solution contained 96 mm NaCl, 2 mm KCl, 1.8 mm CaCl_2_, 2 mm MgCl_2_, and 5 mm HEPES, pH 7.4, supplemented with 50 mg/liter of gentamicin sulfate. Whole cell currents were recorded from oocytes 2–5 days after injection by using the two-electrode voltage-clamp technique. All electrophysiological experiments were performed at a constant temperature (18 °C) by using an Axoclamp 900A amplifier (Molecular Devices) controlled by a pClamp 10.4 data acquisition system (Molecular Devices). Data were sampled at a frequency of 100 Hz and low-pass filtered at 20 Hz by using a four-pole Bessel filter. Voltage and current electrodes were backfilled with 3 m KCl, and their resistances were kept as low as possible (<1 megaohm). Oocytes were clamped at a holding potential of −60 mV throughout all recordings. To minimize interference of endogenous Ca^2+^-activated Cl^−^ channels during the experiments, a Ca^2+^-free bath perfusion solution was used, containing 82.5 mm NaCl, 2.5 mm KCl, 1 mm MgCl_2_, 2.5 mm BaCl_2_, and 5 mm HEPES, pH 7.4. Current responses were evoked by 10-s applications of 200 μm acetylcholine and increasing fragment concentrations, separated by 200-s ligand-free intervals. Concentration-inhibition curves were fitted according to the Hill equation: *y* = *I*_max_/[1 + (IC_50_/[fragment])*^nH^*], where *y* is the normalized current amplitude, *I*_max_ is the maximal current, IC_50_ is the fragment concentration at half-maximal inhibition, [fragment] is the fragment concentration and *n*_H_ is the Hill coefficient. All current responses were analyzed by using Clampfit 10.4 (Molecular Devices), Excel 2007 (Microsoft), and Origin 7.5 (OriginLab). Statistical differences were assessed by using a Student's *t* test.

## Author contributions

C. U., S. L., and J. T. designed the research; F. D., M. B., F. G., S. N., S. P., J. B.-N. performed experiments; F. D., F. G., S. N., S. P., S. B., P. C., J. B.-N., F. v. D., W. G. T., R. P. J., and C. U. analyzed the data; C. U. wrote the paper with contributions from all authors.

## Footnotes

5 Please note that the JBC is not responsible for the long-term archiving and maintenance of this site or any other third party hosted site.

## Footnotes

4 The abbreviations used are:

pLGIC: pentameric ligand-gated ion channel
nAChR: nicotinic acetylcholine receptor
AChBP: acetylcholine-binding protein
GABA_A_Rs: type A γ-aminobutyric acid receptors
GlyRs: glycine receptors
ELIC: *Erwinia* ligand-gated ion channel
GLIC: *Gloeobacter* ligand-gated ion channel
PAM: positive allosteric modulator
α7-AChBP_VS_: α7-acetylcholine–binding protein with additional humanizing mutations in the vestibule site
SPR: surface plasmon resonance
TQS: 4-(1-napthyl)-3*a*,4,5,9*b*-tetrahydro-3*H*-cyclopenta[*c*]quinoline-8-sulfonamide
PNU-120596: 1-(5-chloro-2,4-dimethoxy-phenyl)-3-(5-methyl-isoxazol-3-yl)-urea
NS-1738: 1-(5-chloro-2-hydroxy-phenyl)-3-(2-chloro-5-trifluoromethyl-phenyl)-urea
LY-2087101: [2-(4-fluoro-phenylamino)-4-methyl-thiazol-5-yl]-thiophen-3-yl-methanone
CU2017: *N*-(5-bromopyridin-2-yl)piperidine-3-carboxamide
PDB: Protein Data Bank.
